# Oral Administration of Oleuropein and Its Semisynthetic Peracetylated Derivative Prevents Hepatic Steatosis, Hyperinsulinemia, and Weight Gain in Mice Fed with High Fat Cafeteria Diet

**DOI:** 10.1155/2015/431453

**Published:** 2015-12-22

**Authors:** Saverio Massimo Lepore, Valeria Maria Morittu, Marilena Celano, Francesca Trimboli, Manuela Oliverio, Antonio Procopio, Carla Di Loreto, Giuseppe Damante, Domenico Britti, Stefania Bulotta, Diego Russo

**Affiliations:** ^1^Department of Health Sciences, University “Magna Graecia” of Catanzaro, Campus “S. Venuta”, Viale Europa, Germaneto, 88100 Catanzaro, Italy; ^2^Department of Medical and Biological Sciences, University of Udine, Piazzale Kolbe 4, 33100 Udine, Italy

## Abstract

The high consumption of olive tree products in the Mediterranean diet has been associated with a lower incidence of metabolic disorders and cardiovascular diseases. In particular, the protective effects of olive oil have been attributed to the presence of polyphenols such as oleuropein (Ole) and its derivatives. We have synthesized a peracetylated derivative of Ole (Ac-Ole) which has shown* in vitro* antioxidant and growth-inhibitory activity higher than the natural molecule. In this study, male C57BL/6JOlaHsd mice were fed with a standard (std), cafeteria (caf) diet, and caf diet supplemented with Ole (0.037 mmol/kg/day) and Ac-Ole (0.025 mmol/kg/day) for 15 weeks. We observed a significant reduction in the caf diet-induced body weight gain and increase of abdominal adipose tissue. Also, Ole and Ac-Ole prevented the development of hepatic steatosis. Finally, Ole and Ac-Ole determined a lower increase of HDL and LDL-cholesterol levels and corrected caf diet-induced elevation of plasma glucose concentrations by improving insulin sensitivity. The observed beneficial properties of Ole and Ac-Ole make these compounds and in particular Ac-Ole promising candidates for a potential pharmaceutic use in metabolic disorders.

## 1. Introduction

Dietary consumption of olive oil is considered a key component to explain the association of Mediterranean diet with a lowered incidence of metabolic disorders and cardiovascular diseases [1–3]. The beneficial action of extra virgin olive oil is mainly attributed to its seco-phenolic compounds and their secondary metabolites, which have highly been investigated as both whole extracts and individual components [4, 5]. In particular, the proactive ingredient oleuropein (Ole) and its derivative hydroxytyrosol (HT) have demonstrated several beneficial effects in* in vitro* and* in vivo* experimental models [6–9]. Ole is a natural polyphenolic compound belonging to the secoiridoids and is present in high amount in the leaves and unprocessed olive drupes. After crushing, Ole is hydrolyzed by endogenous esterase giving rise to its aglycone derivate that is the active secoiridoid present in virgin olive oil [8]. After oral administration, it is rapidly absorbed with maximum plasma concentration occurring 2 h after administration and excreted in urine mainly as glucuronides or as free form in very low concentrations [10, 11].

The ability of Ole and Ole derivatives to scavenge reactive oxygen species and their antioxidant activity [6, 8, 12, 13] have been associated with the protection against cardiovascular diseases and metabolic disorders [14–17]. Two other mechanisms have been proposed to explain the hypoglycemic effect of Ole and HT: the improvement of glucose-induced insulin release and the increased peripheral uptake of glucose [14, 18]. Moreover, an effect of Ole on insulin action and secretion has been described in overweight middle-aged men, who received a diet supplemented with olive leaf polyphenols. Such an effect was independent by fat distribution, dietary intakes, and physical activity and was similar to that obtained with antidiabetic drug treatment [19]. In addition, Ole has been successfully used to prevent hepatic steatosis in mice fed with high fat diet [9, 20–22].

In our laboratory, we have synthesized a peracetylated derivative of Ole (Ac-Ole) in good yields and very mild conditions by applying nonconventional synthetic methodologies on the natural substrate Ole extracted from renewable raw material [23, 24]. In previous works, Ac-Ole has shown* in vitro* higher antioxidant and antiproliferative effects on thyroid and breast cancer cells than the natural molecule [25, 26].

A relevant model to study metabolic disorders in mice is the highly palatable cafeteria (caf) diet: this dietary intervention is closer to Western food habits than the traditional high fat diet, creating therefore a phenotype of exaggerated obesity with glucose intolerance and inflammation [27].

In the present study, we analyzed the effects of oral subchronic administration of Ole and Ac-Ole in C57BL/6JOlaHsd mice fed with a caf diet.

## 2. Methods

### 2.1. Diet Composition and Compounds

The nutritional compositions of the standard (std) diet (Teklad Global 18% Protein Rodent Diet, Harlan Laboratories s.r.l., Udine, Italy) and the caf diet are reported in Table 1. Caf diet is a mixture of std diet and commercial food as snack, chocolate, biscuits, and wafers. Ole (MW 540, dissolved in water at the dose of 0.037 mmol/kg, equivalent to 20 mg/kg) and its semisynthetic derivative Ac-Ole (MW 792, dissolved in corn oil at the dose of 0.025 mmol/kg, equivalent to 20 mg/kg) were obtained as described [23].

### 2.2. Animals and Treatment

Male C57BL/6JOlaHsd mice (*n* = 20, aged 4 weeks) were purchased from Harlan Laboratories s.r.l., housed in a temperature (20–22°C) and humidity (64%) controlled animal room and maintained on a 12 : 12 h light/dark cycle. After 2 weeks of quarantine the mice were divided into four separate groups (*n* = 5 in each group) of equal average body weight (19.4 ± 1.2 g): mice fed with a std diet (named STD), mice fed with a caf diet (named CAF), mice fed with a caf diet supplemented with daily oral administration of Ole (0.037 mmol/Kg; named OLE), and mice fed with a caf diet supplemented with daily oral administration of Ac-Ole (0.025 mmol/Kg; named Ac-OLE). A std diet (Harlan Laboratories s.r.l.), suggested for this strain of mice, was used as control to (a) analyze the effects of caf diet and (b) investigate the effects of treatment with Ole and Ac-Ole. Animals of all groups received the same vehicle used for the peracetylated compound and had free access to food and water. Every day, Ole and Ac-Ole were prepared freshly before administration and added to caf diet pellet: it was checked that each mouse had ingested the food containing the daily dose of Ole and Ac-Ole. Food intake was calculated subtracting every week the aliquot of food not ingested by the total food added to each cage. Body weight, nasoanal (N-A) length, and girth waist of all animals were recorded at weekly interval. At the end of treatment, after overnight fasting, mice were deeply anesthetized by intraperitoneal injection of tiletamine hydrochloride and zolazepam hydrochloride (Zoletil, Virbac, France) at dosage of 80 mg/kg, and medetomidine hydrochloride (Domitor, Orion Corporation, Finland) at dosage of 6.6 mg/kg. Blood samples were obtained by cardiac venipuncture and collected into blood collection tubes (Vacuette, Greiner Bio-One GmbH Bad Haller Str. 32, 4550 Kremsmunster, Austria) containing K_3_-EDTA. The whole liver, abdominal fat, thoracic aorta, kidney, and lung were removed and weighed and then were washed in normal saline solution and immediately fixed in 10% neutral buffered formalin (Sigma-Aldrich s.r.l., Milan, Italy). All efforts were made to minimize the number of animals used in this study and their suffering.

### 2.3. Biochemical Analysis

Blood samples, collected at the end of the treatments, were centrifuged at 1700 g for 10 min at room temperature and serum was stored at −20°C until use. By using commercial reagents (Siemens Healthcare Diagnostics s.r.l., Milan, Italy) and an automated biochemistry analyzer (Dimension EXL, Siemens Healthcare Diagnostics s.r.l.) the levels of basal glucose, triglycerides, total cholesterol, low density lipoproteins cholesterol (LDL), high density lipoproteins cholesterol (HDL), alanine aminotransferase (ALT), and aspartate aminotransferase (AST) were determined. The concentration of insulin and leptin was measured using ELISA kits (Rat/Mouse Insulin ELISA Kit; Mouse Leptin ELISA Kit, EMD Millipore Corporation, Darmstadt, Germany) according to the manufacturers' instructions. Approximate insulin resistance (IR) was calculated using the homeostasis model assessment (HOMA)-IR using the following formula: [glucose (mmol/L) × insulin (*μ*U/mL)]/22.5 [28].

### 2.4. Histopathological Study

Tissue samples were embedded in paraffin after dehydration through a graded ethanol series followed by xylene. Several sections (thickness 4-5 *μ*m) from each paraffin block were cut, mounted on slides and, after deparaffinization and rehydration, were stained with haematoxylin and eosin according to standard methods for histological assessment under the light microscopy. In liver of each mouse, steatosis was evaluated by counting the percentage of vacuolated cells. For each liver two different sections were evaluated, each of them cut along the major axis of the gland. All cells of each section were evaluated. The Gomori trichrome stain was used to detect fibrosis [29].

### 2.5. Statistical Analysis

Results are expressed as mean ± standard deviation (SD). The data relative to body weight, naso-anal length, girth waist, food consumption, energy intake, liver weight, and hematological and clinical chemistry parameters were analyzed using Sigma Plot version 12 (Systat Software, Inc.). One-way ANOVA followed by Tukey multiple comparison test was performed for different treatment groups. Differences between groups were considered significant at *P* < 0.05.

## 3. Results

### 3.1. Ole and Ac-Ole Prevented Increase in Body and Abdominal Fat Weight

**Figure 1:**
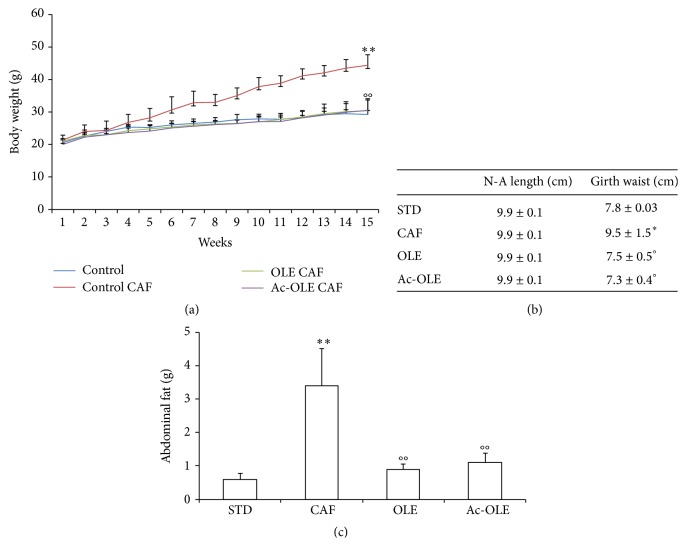
Effects of Ole and Ac-Ole on body weight and girth waist. (a) Body weight and (b) N-A length and girth waist are relative to the end of treatment (15 weeks). (c) Abdominal fat tissue weight. Values are mean ± SD. ^*∗*^ *P* < 0.05 and ^*∗∗*^ *P* < 0.01 compared to mice fed with std diet; °*P* < 0.05 and °°*P* < 0.01 compared to mice fed with caf diet.

Mice were fed for 15 weeks with std, caf, or caf diet supplemented with Ole or Ac-Ole. During the experimental period, the mice showed no signs of suffering and no significant differences appeared in hematological parameters at the end of the treatments (Table 2). The body weight increased in all groups, in accordance with the physiological growth of the mice (Figure 1(a)). In CAF mice, this increase was significantly higher than STD (Figure 1(a), *P* < 0.01), in parallel with an enhanced abdominal girth (Figure 1(b), *P* < 0.05) and an increase in abdominal fat (Figure 1(c), *P* < 0.01). Supplementation of caf diet with Ole or Ac-Ole reduced the body weight increase (Figure 1(a), *P* < 0.01), as well as both abdominal girth and final abdominal fat (Figures 1(b) and 1(c), *P* < 0.05 and *P* < 0.01, resp.). The food intake did not differ among the four groups during the experimental period, whereas a significant increase of energy intake was produced by caf diet (Table 3, *P* < 0.001), and the addition of the two compounds did not cause significant changes in OLE and Ac-OLE-treated groups when compared to CAF group (Table 3).

### 3.2. Ole and Ac-Ole Treatment Prevented Lipid Accumulation in Liver and Other Tissues

**Figure 2:**
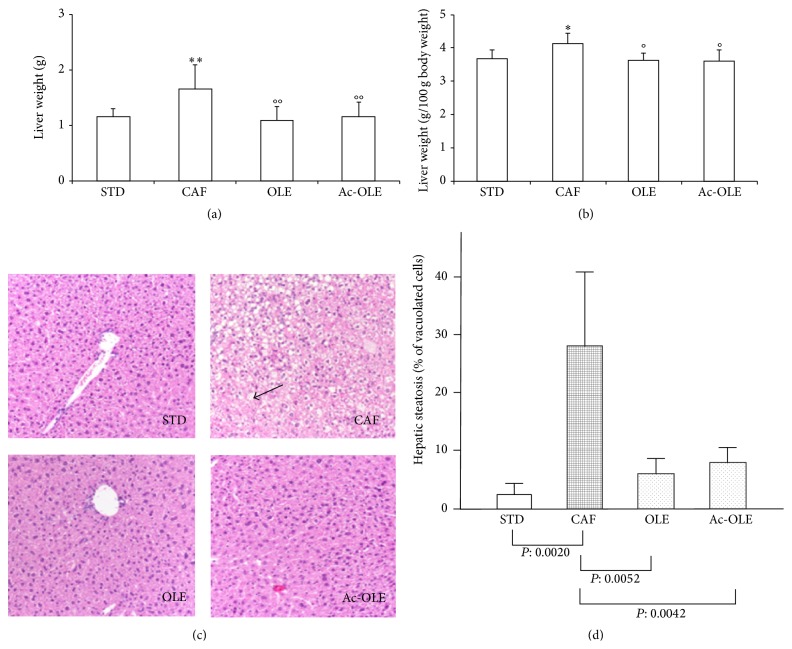
Effects of Ole and Ac-Ole on liver weight and liver histology. (a) Liver weight was significantly reduced in mice fed with a caf diet supplemented with Ole or Ac-Ole. (b) Relative liver weight. (c) Hematoxylin and eosin staining of liver sections (10x magnification). The liver of mice CAF was rich in fat vacuoles (indicated by the arrow). (d) Quantification of steatosis was expressed as mean of percentage of vacuolated cells calculated for each mouse liver. Values are mean ± SD. ^*∗*^ *P* < 0.05 and ^*∗∗*^ *P* < 0.01 compared to STD; °*P* < 0.05 and °°*P* < 0.01 compared to CAF.

**Figure 3:**
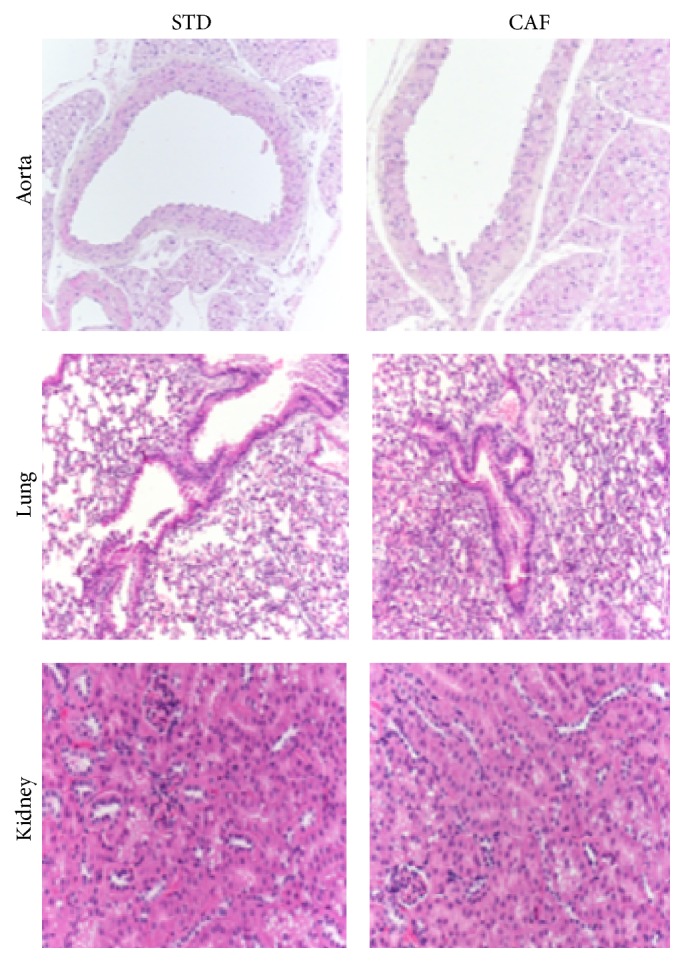
Histologic analysis of aorta, lung, and kidney. Hematoxylin and eosin staining of aorta, lung, and kidney sections (10x magnification) from mice fed with std or caf diet.

Liver weight was significantly increased in CAF (1.6 ± 0.4 g) versus STD (1.1 ± 0.1 g). Treatment with Ole and Ac-Ole prevented hepatic enlargement, as evident by the weight values, both absolute and normalized for the body weight, similar to those of the STD mice (Figures 2(a) and 2(b)). Histologic analysis of liver tissue sections representative of the four groups of animals is shown in Figure 2(c) and the quantitation of steatosis is reported in Figure 2(d): caf diet induced a significant steatosis, which was greatly reduced in OLE and Ac-OLE groups. In terms of inflammatory cell infiltration, no significant difference was detectable among the four groups of animals; indeed, in all mice only a mild and very focal lymphocytic infiltration in the portal triad was detectable. Gomori staining indicates the absence of fibrosis in all four groups of animals (data not shown). Caf diet induced no detectable differences in the other examined tissues (Figure 3).

### 3.3. Effects of Ole and Ac-Ole on Serum Lipid Levels

Caf fed mice showed significantly higher levels of plasma total, HDL, and LDL cholesterol than the std fed mice (Table 4). Ole and Ac-Ole supplemented caf diet determined a lower increase of such levels and, interestingly, LDL-cholesterol levels were maintained in the normal range described for these animals. No significant differences were observed in the levels of plasmatic triglycerides (Table 4). In addition, caf diet induced an elevation in ALT and AST levels when compared with STD group. Following treatment with Ole, only the ALT levels returned to the values of the STD, while Ac-Ole determined a reduction of both enzymes (Table 4). However, transaminase serum levels remained in the normal range in all four groups of mice.

### 3.4. Ole and Ac-Ole Reduced Glucose, Insulin, and Leptin Levels as well as Insulin Resistance Induced by Caf Diet

**Figure 4:**
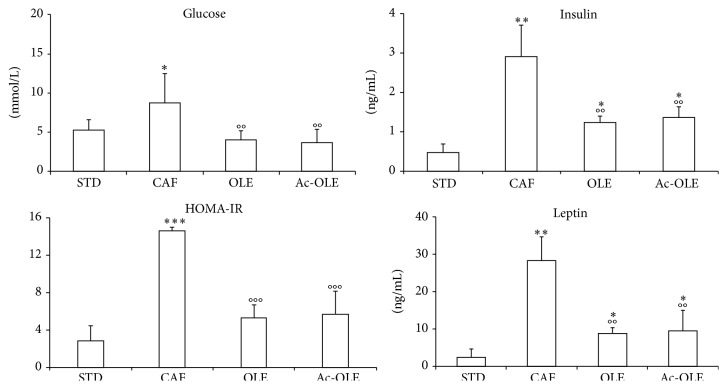
Effects on plasma levels of glucose, insulin, and leptin and evaluation of insulin resistance after treatment with Ole and Ac-Ole. Blood samples were collected as indicated in Methods. Glucose and hormones levels were significantly lower after treatment with Ole and Ac-Ole in animals fed with caf diet. Homeostasis model assessment of insulin resistance (HOMA-IR) was calculated as indicated in Methods. Values are mean ± SD. ^*∗*^ *P* < 0.05, ^*∗∗*^ *P* < 0.01, and ^*∗∗∗*^ *P* < 0.001 compared to STD; °°*P* < 0.01 and °°°*P* < 0.001 compared to CAF.

Fasting glucose levels were slightly but significantly higher in CAF animals compared to mice fed with std diet, although still in the normal range (Figure 4). Instead, OLE and Ac-OLE groups showed values similar to STD (Figure 4). Interestingly, insulin levels were significantly higher in CAF mice as compared to STD group (Figure 4, *P* < 0.01), while, in both OLE and Ac-OLE animals, they were significantly lower compared to the CAF group (Figure 4, *P* < 0.01). HOMA-IR, used as measure of insulin sensitivity, indicated that CAF group showed statistically significant increase in insulin resistance compared to STD group (Figure 4, *P* < 0.001) while the supplementation of Ole and Ac-Ole on caf diet reversed this effect (Figure 4, *P* < 0.001 versus CAF). Also leptin content was significantly changed in CAF as compared to STD mice (Figure 4, *P* < 0.01); again, lower levels were observed in the groups treated with Ole or its peracetylated derivative (Figure 4, *P* < 0.01 versus CAF).

## 4. Discussion

Polyphenolic compounds of extra virgin olive oil are known to exert a series of beneficial effects, mainly due to their antioxidant activity. A large number of studies that performed* in vitro* and* in vivo* experimental models have focused on the activity of Ole revealing a large array of biological effects either when used as single compound or in a mixture of phenolic extracts from olive oil drupes or leaves [4, 6, 7, 17]. In view of a possible use in pharmaceutical field as single drug or as additive in the food, we have synthesized and tested in our laboratory a peracetylated derivative of Ole [23, 25, 26]. Peracetylation and deacetylation occur naturally in cells thanks to the activity of mitochondrial acetyl-CoA dependent enzymes [30–32]. It has been demonstrated that unprotected drugs, such as N-substituted aromatic compounds, are N-acetylated inside the cells in order to limit their adverse biological effects [30]. Similarly, peracetylated natural compounds such as peracetylated resveratrol or peracetylated epigallocatechin-3-gallate can act as prodrugs because they are deacetylated inside the cells [33]. Since peracetylation improves the chemical stability and permeability without affecting the transportation pathway through the membrane, the intracellular deacetylation results in an augmented dose of unprotected natural active principle in the intracellular medium [25, 26, 32].

We have previously reported the beneficial effects of Ole and Ac-Ole* in vitro*. In this study, they have been investigated* in vivo* to analyze the effects versus the metabolic alterations caused by a caf diet. This diet is composed of a mixture of fat and carbohydrates and resembles in a better way the Western type food habits compared to traditional lard-based high fat diet [27]. Indeed, mice fed with a caf diet develop metabolic syndrome more severely than those fed with high fat diet [34].

In our study, mice fed with this diet for 15 weeks presented an increased body weight associated with histological signs of hepatic steatosis, high serum levels of total and LDL-cholesterol and hyperinsulinemia. Only slight changes and inside the normal range levels were observed for transaminases, probably due to the limited period of exposure to this diet, as already reported in the other study [22]. Interestingly, the presence in CAF mice of an increase in insulin levels seems to reproduce the well-known phase of hyperinsulinemic normoglycemia observed in obese individuals before the development of diabetes mellitus 2 (DM2). In our caf fed mice, administration of 0.037 mmol/kg/day of Ole and 0.025 mmol/kg/day of Ac-Ole, determined the following: (a) reduction in the body weight increase due to minor accumulation of fat in the abdominal adipose tissue, with unchanged food intake gain; (b) significant decrease of macroscopic and microscopic steatosis; (c) lower levels of serum lipids, with normalization of the levels of LDL-cholesterol; (d) correction of elevated glucose plasmatic levels without increase in insulin levels, associated with normal HOMA-IR.

In a recent study, van der Stelt et al. [35] demonstrated that high dose of Ole supplementation (758 mg/kg body weight) in mice fed with a high fat diet resulted in decrease of body weight, serum lipids, hepatic lipid accumulation, and liver weight after 12 weeks of treatment. In our study, the same results were observed using a much lower dose of Ole or Ac-Ole (20 mg/kg body weight). Prevention of body weight increase in animals (mice or rats) fed with high fat diet without acting on food intake was also reported using a similar dosage of Ole in similar experimental conditions [36, 37]. As in our study, no action on food intake was observed when Ole was added as single compound, while a decrease was detected when olive leaf phenolic extracts were tested [38], probably owing to the presence of apigenin [39, 40]. Also, the caloric intake of OLE and Ac-OLE groups was significantly higher with respect to STD but they did not gain weight; we may hypothesize that Ole and Ac-Ole act by increasing the energy expenditure and/or reducing energy retention, by regulating the expression of genes involved in adipogenesis and thermogenesis [38]. Our present data showed that the same prevention of body weight increase was obtained with lower doses of Ac-Ole (0.025 versus 0.037 mmol/kg of Ole). Further studies on the gene expression profile modified by both compounds will help to identify the molecular pathways involved in such an effect. Also prevention of hepatic steatosis exerted by Ole has already been described in animal models [21, 22]. As for the protective action on body weight increase, an identical effect was obtained in our experimental model using Ac-Ole. Again, as for the anti-flogistic and* in vitro* antineoplastic effects [25, 26, 41, 42], this semisynthetic compound demonstrated high efficacy.

The most interesting findings of the present study, in our opinion, appear to be those regarding the metabolic effects of the compounds. In our experimental model, exposure to a caf diet determined a significant increase in both total and LDL-cholesterol levels. Ole and Ac-Ole treatment was able to partially prevent such effects and, most importantly, normalize the LDL-cholesterol levels. Andreadou et al. [43, 44] demonstrated that Ole (20 mg/kg) decreased cholesterol and triglycerides levels in hypercholesterolemic rabbits. Instead, we found no significant changes in triglycerides, probably because in this strain of mice treatments longer than 15 weeks are required to get such alteration [22].

Absence of significant modifications was noted in caf fed mice regarding the glycemic profile. However, insulin levels resulting are higher than mice fed with std diet, so that we may suppose that it was the parallel increase of insulin secretion which counteracted the effects of caf diet. Treatment with Ole and Ac-Ole obtained the same result with levels of insulin maintained in the normal range, with normalization of the values of HOMA-IR. In the absence of euglycemic hyperinsulinemic clamp, it is not possible to fully demonstrate the occurrence of an increased insulin sensitivity, as postulated in other studies using olive leaf polyphenols [19]. Another possibility is that normal insulin levels depend on the lack of body weight increase. In any case, however, we believe that prevention of damage associated with prolonged exposure to hyperinsulinemia that characterizes the pathologies associated with metabolic syndrome, DM2, and obesity deserves to be underlined. An interference between Ole (or Ac-Ole) and substances present in elements of caf diet (as well as any kind of commercial diet used in this kind of studies) cannot be excluded.

## 5. Conclusions

In conclusion, these findings demonstrate, in this experimental model of metabolic damage caused by caf diet, the beneficial effects of Ac-Ole, exerted at even lower dose in moles than Ole. Further studies, including the administration of different dosages of Ac-Ole and even for shorter periods will allow characterizing the molecular mechanism involved in such an action, in view of a potential use of this compound for prevention of human metabolic disorders.

## Figures and Tables

**Table 1:** Composition of the standard and cafeteria diets.

	STD	CAF
Energy density (kcal/g)	3.1	4.9

Protein (%)	18.6	8.4

Fat (%) of which saturated (%)	6.2 0.9	26.0 14.8

Carbohydrate (%) of which sugar (%)	44.2 —	52.7 21.5

Fiber (%)	3.5	1.8

Salt (%)	0.2	0.3

Iron (%)	0.02	0.004

Calcium (%)	1.0	0.2

Potassium (%)	0.6	0.2

Phosphorus (%)	0.7	0.2

Zinc (%)	0.007	0.001

B1 (%)	0.0017	0.0003

B2 (%)	0.0015	0.0003

**Table 2:** Effects of Ole and Ac-Ole on hematological parameters.

Parameter	Units	STD	CAF	OLE	Ac-OLE	Range
WBC	(10^3^/mm^3^)	2.4 ± 0.5	2.8 ± 0.8	3.8 ± 1.2	4.0 ± 1.0	2.3–7.7
RBC	(10^6^/mm^3^)	8.6 ± 1.1	9.6 ± 0.2	9.0 ± 0.3	9.3 ± 0.4	8.4–10.2
HGB	(g/dL)	13.4 ± 0.2	14.1 ± 0.5	12.4 ± 0.4	12.8 ± 0.4	12.6–14.6
HCT	(%)	41.9 ± 6.3	46.5 ± 2.1	43.7 ± 1.5	44.3 ± 2.1	41.9–49.9
MCV	(fL)	48.3 ± 1.2	48.3 ± 1.5	48.4 ± 1.1	47.4 ± 0.6	48.0–52.0
MCH	(pg)	14.6 ± 0.1	14.7 ± 0.4	13.8 ± 0.2	13.7 ± 0.1	14.3–15.3
MCHC	(%)	30.4 ± 0.7	30.4 ± 0.5	28.4 ± 0.4	29.0 ± 0.4	28.6–30.8
Platelets	(10^3^/*μ*L)	1183.2 ± 71.4	1097.0 ± 316.8	1170.2 ± 160.0	1041.2 ± 317.7	980–1390
Neutrophils	(%)	7.4 ± 1.8	13.1 ± 6.4	9.8 ± 1.8	11.4 ± 2.1	2–34
Lymphocytes	(%)	87.6 ± 1.1	81.4 ± 7.3	86.2 ± 2.3	80.9 ± 5.6	59–97
Monocytes	(%)	0.8 ± 0.2	1.4 ± 0.9	1.0 ± 0.2	1.3 ± 1.7	0–6
Eosinophils	(%)	1.6 ± 0.5	2.1 ± 0.8	1.4 ± 0.8	2.3 ± 1.4	0–2

Blood samples were collected at the end of treatments and analysed using Hematology Analyzer ADVIA 2120 (Siemens Healthcare Diagnostics s.r.l., Milan, Italy) to obtain hematology parameters. STD: mice fed with STD diet; CAF: mice fed with CAF diet; OLE = mice fed with CAF diet plus OLE at 0.037 mmol/kg/day; Ac-OLE: mice fed with CAF diet plus Ac-OLE at 0.025 mmol/kg/day. WBC: white blood cells; RBC: red blood cells; HGB: hemoglobin; HCT: hematocrit; MCV: mean corpuscular volume; MCH: mean corpuscular hemoglobin; MCHC: mean corpuscular hemoglobin concentration.

**Table 3:** Effects of Ole and Ac-Ole on food and energy intake.

	STD	CAF	OLE	Ac-OLE
Food intake (g/week)	20.8 ± 0.8	22.8 ± 2.8	22.1 ± 2.4	21.6 ± 2.1
Energy intake (kcal/week)	64.5 ± 2.6	103.0 ± 9.2^*∗∗∗*^	101.2 ± 10.8^*∗∗∗*^	99.9 ± 6.2^*∗∗∗*^

STD: mice fed with STD diet; CAF: mice fed with CAF diet; OLE = mice fed with CAF diet plus Ole at 0.037 mmol/kg/day; Ac-OLE = mice fed with CAF diet plus Ac-Ole at 0.025 mmol/kg/day. Values are mean ± SD. ^*∗∗∗*^ *P* < 0.001 versus STD.

**Table 4:** Effects of Ole and Ac-Ole on serum parameters.

Parameter	Units	STD	CAF	OLE	Ac-OLE	Range
Cholesterol	mg/dL	108.7 ± 6.2	183.0 ± 13.4^*∗∗∗*^	158.2 ± 23.4^*∗∗*^	158.5 ± 18.5^*∗∗*^	43.7–126.2
HDL	mg/dL	94.7 ± 3.8	136.0 ± 7.5^*∗∗*^	121.0 ± 12.3	123.2 ± 19.5^*∗*^	51.1–102.5
LDL	mg/dL	14.0 ± 2.7	47.0 ± 7^*∗∗∗*^	29.3 ± 4.7^*∗∗*°°^	30.7 ± 3.4^*∗∗*°°^	13.8–31.0
Triglycerides	mg/dL	47.0 ± 8.9	52.6 ± 3.5	39.0 ± 6.5	57.5 ± 6.5	47.1–254.9
ALT	U/L	36.5 ± 1.9	41.7 ± 2.2^*∗*^	28.7 ± 0.9°°°	31.0 ± 7.8°°°	13.3–80.2
AST	U/L	97.7 ± 11.1	169.3 ± 10.7^*∗∗∗*^	128.8 ± 7.4°	90.0 ± 8.5°°°	40.8–510.6

STD: mice fed with STD diet; CAF: mice fed with CAF diet; OLE: mice fed with CAF diet plus Ole at 0.037 mmol/kg/day; Ac-OLE: mice fed with CAF diet plus Ac-Ole at 0.025 mmol/kg/day; ALT: alanine aminotransferase; AST: aspartate aminotransferase; HDL: high density lipoproteins; LDL: low density lipoproteins. Reference range calculated on the data published in Mouse Database Phenomena (Jackson Laboratory) (http://phenome.jax.org). Values are mean ± SD. ^*∗*^ *P* < 0.05, ^*∗∗*^ *P* < 0.01, and ^*∗∗∗*^ *P* < 0.001 compared to STD; °*P* < 0.05, °°*P* < 0.01, and °°°*P* < 0.001 compared to CAF.
